# Study of therapeutic hypothermia (32 to 35°C) for intracranial pressure reduction after traumatic brain injury (the Eurotherm3235Trial): outcome of the pilot phase of the trial

**DOI:** 10.1186/1745-6215-14-277

**Published:** 2013-09-03

**Authors:** Peter J D Andrews, Louise H Sinclair, Bridget Harris, Melissa J Baldwin, Claire G Battison, Jonathan K J Rhodes, Gordon Murray, Daniel De Backer

**Affiliations:** 1 Department of Anaesthesia and Pain Management, University of Edinburgh, Edinburgh, UK; 2 NHS Lothian, Waverley Gate, 2-4 Waterloo Place, Edinburgh EH1 3EG, UK; 3 Centre for Population Health Sciences, The University of Edinburgh, Medical School, Teviot Place, Edinburgh EH8 9AG, UK; 4 Erasme University Hospital, Free University of Brussels, 808 Route de Lennick, Brussels B-1070, Belgium

**Keywords:** Traumatic brain injury, Therapeutic hypothermia, Randomized controlled trial, Feasibility

## Abstract

**Background:**

Clinical trials in traumatic brain injury (TBI) are challenging. Previous trials of complex interventions were conducted in high-income countries, reported long lead times for site setup and low screened-to-recruitment rates.

In this report we evaluate the internal pilot phase of an international, multicentre TBI trial of a complex intervention to assess: design and implementation of an online case report form; feasibility of recruitment (sites and patients); feasibility and effectiveness of delivery of the protocol.

**Methods:**

All aspects of the pilot phase of the trial were conducted as for the main trial. The pilot phase had oversight by independent Steering and Data Monitoring committees.

**Results:**

Forty sites across 12 countries gained ethical approval. Thirty seven of 40 sites were initiated for recruitment. Of these, 29 had screened patients and 21 randomized at least one patient. Lead times to ethics approval (6.8 weeks), hospital approval (18 weeks), interest to set up (61 weeks), set up to screening (11 weeks), and set up to randomization (31.6 weeks) are comparable with other international trials. Sixteen per cent of screened patients were eligible. We found 88% compliance rate with trial protocol.

**Conclusion:**

The pilot data demonstrated good feasibility for this large international multicentre randomized controlled trial of hypothermia to control intracranial pressure. The sample size was reduced to 600 patients because of homogeneity of the patient group and we showed an optimized cooling intervention could be delivered.

**Trial registration:**

Current Controlled Trials: ISRCTN34555414.

## Background

The pattern of severe traumatic brain injury (TBI) is changing globally with the incidence increasing rapidly in low- and middle-income countries as a result of increased motor vehicle use [1]. Previous trials of complex interventions in TBI patients have been conducted in high-income countries such as Europe and the US. However, the increasing cost and administrative burden of conducting large clinical trials has forced researchers to conduct trials in low-income countries where costs are lower and the potential patient pool is higher [2]. If treatments for TBI patients are to be successful, future trials of any intervention that may improve outcome after TBI should be tested in high-, middle- and low-income countries to assess generalizability [2].

A report published by the European Forum for Good Clinical Practice [3] aimed to evaluate the impact of Directive 2001/20/EC, the Clinical Trial Directive throughout Europe. Results of the survey showed a 30% extended timeline to protocol approval as a result of increased workload for Ethics Committees, less harmonization of the ethical review process, variable costs and review timeframes between countries, and the added complexity of the trial Sponsor contracting insurance for participating hospitals. A proposal to amend the Directive is currently with the European Parliament in an attempt to streamline and improve practices throughout the European Union.

Clinical trials in severe TBI are therefore challenging due to global incidence, administrative burden associated with large clinical trials and the heterogeneity of the condition. Previous trials of TBI patients have reported long lead times for site setup/approvals and low screened-to-recruitment rates [4]. A balance must therefore be achieved between our current understanding of the mechanisms of action of the intervention being evaluated and maximizing recruitment by using broad inclusion criteria.

The National Institute of Neurological Disorders and Stroke determined in October 2007 that recruitment of heterogeneous TBI patients to clinical trials was a major impediment to success [5]. Clinical trials using broad inclusion criteria could, however, use a statistical analysis plan incorporating a pre-specified covariate adjustment to reduce the effects of heterogeneity [6].

Current treatments for TBI emphasize restoring and maintaining adequate brain perfusion, surgically evacuating large hematomas where necessary, and preventing or promptly treating edema [7]. Brain swelling can be monitored by measuring intracranial pressure (ICP) and, in most centers, ICP is used to guide treatments and to monitor their success [8]. The initiation of hypothermia following severe TBI is used in this context despite few supporting data. However, the need for resuscitation and computerized tomography imaging to confirm the diagnosis in patients with TBI are factors which delay intervention with temperature reduction strategies [9].

The use of hypothermia to improve outcomes in patients with acute brain injury is not a new idea. However, the evidence to date of efficacy from prophylactic neuroprotection trials in patients with TBI is modest [7,10]. The Eurotherm3235Trial is an international, multicentre, randomized controlled trial investigating if therapeutic hypothermia (32 to 35°C) used to manage raised ICP following severe TBI leads to improved outcome. No trial has previously tested this treatment paradigm.

### Study objectives

The Eurotherm3235Trial aims to answer the research question: does titrated hypothermia to reduce raised ICP after TBI improve outcome at 6 months?

In this report we evaluate data collected during the internal pilot phase (January 2009 to August 2011) to assess the following criteria: design and implementation of an online electronic case report form (eCRF) for screening, randomisation and data collection; feasibility of recruitment (sites and patients); patient eligibility - previous observational studies had predicted that 50% of all TBI patients would be eligible [11]; and feasibility of the protocol, in particular the effectiveness of delivery of the cooling protocol.

## Methods

The trial protocol was developed by the Edinburgh trial team in collaboration with an international advisory board that included key experts in this field of research, experienced trialists, specialist nurses and statisticians [12].

Full details of the trial protocol have been published previously [12]. In summary, this is a pragmatic, multicentre randomized controlled trial examining the effects of hypothermia (32 to 35°C), titrated to reduce ICP to below 20 mmHg, on morbidity and mortality 6 months after TBI (extended Glasgow Outcome Scale (GOSE)). The study aimed to recruit 1,800 patients over 41 months. Enrolment started in November 2009.

The main ethical issue with this trial is that patients are adults with incapacity. We have complied with the devolved nations legal requirements and have favorable ethical opinion from all (09/MRE00/34; Scotland A research Ethics Committee). Similar arrangements have been satisfied in another 13 countries. Due to their incapacitated state, it is not be possible to obtain written consent directly from potential participants. Consent is therefore sought from each eligible patient’s nearest relative or another person designated to give consent on the patient’s behalf.

Participants are randomized to either standard care or standard care with titrated therapeutic hypothermia. Hypothermia is initiated with 20 to 30 ml/kg intravenous refrigerated 0.9% sodium chloride and maintained using each site’s usual cooling technique. There is a guideline for detection and treatment of shivering in the intervention group. Hypothermia is maintained for at least 48 hours in the treatment group and continued for as long as is necessary to maintain ICP <20 mmHg. Intracranial hypertension is defined as an ICP >20 mmHg in accordance with the Brain Trauma Foundation Guidelines, 2007 [7].

The Eurotherm3235Trial cooling protocol aims to lower core temperature by the minimum required to control ICP <20 mmHg, within the limits of 32 to 35°C. If a patient’s ICP is not controlled on cooling to 35°C, core temperature is lowered in 0.5°C increments until ICP is <20 mmHg, to a minimum temperature of 32°C. Therapeutic hypothermia is maintained for a minimum of 48 hours.

All aspects of the pilot phase of the trial were conducted as for the main trial. The pilot phase of the trial had oversight by an independent Data Safety Monitoring Board (DSMB) who work according to a charter (based upon DAMOCLES principles) signed by all three members (Peter Suter, Ian Ford and Kathy Rowan). They report to an independent Trial Steering Committee and recommend whether the trial should continue recruiting. The DSMB charter allows for stopping if there is overwhelming evidence of benefit or harm (this or another trial) but no other interim analysis is planned. The funder (European Society of Intensive Care Medicine) appointed a “Research, Scientific and Financial Committee” providing further oversight.

## Results

### Design and implementation

Systems for trial screening, randomization and data management were developed between January 2009 and June 2009. The first patients were randomized in Edinburgh between November 2009 and February 2010, testing the protocol and the paper case record form. Thereafter the online randomization service (minimization with a random element) and electronic data collection database were introduced and validated. Recruitment using this system started in July 2010. Up to the end of August 2011, 416 patients were screened and 67 randomized to the trial.

In keeping with a pragmatic trial design, the protocol and data collection were not burdensome. We collected hourly data on ICP management and core temperature. This allowed assessment of whether sites were able to manage therapeutic hypothermia according to the protocol, including controlled rewarming, and monitor the management of the control group. Crossovers from standard care (control group) to hypothermia were recorded.

### Feasibility of recruitment

During the pilot phase of the trial, 40 sites across 12 countries gained ethical approval (sites per country): Scotland (3), England (11), Northern Ireland (1), Belgium (8), Ireland (1), Italy (4), Germany (2), Greece (6), Estonia (1), Hungary (1), Russia (1) and India (1). Ethical applications were also submitted for a further 9 sites: Brazil (3), Italy (1), Portugal (1) and Spain (4).

Thirty seven of the 40 sites with ethical approval were initiated for recruitment. Of these, 29 had screened patients and 21 randomized at least one patient during the pilot phase.

Across the 37 initiated sites, 416 patients were logged on the eCRF database. Of these, 67 were randomized. The average time from set up to screening was 11 weeks and the time from screening to first randomized patient was 20 weeks. Follow-up for 6 month outcome during the pilot phase was 100%. Table 1 shows site set up times during the pilot phase of the Eurotherm3235Trial.

**Table 1:** Site set up times during the pilot phase of the Eurotherm3235Trial

**Average time taken per country**	**Time to ethics approval (weeks)**	**Time to hospital approval (weeks)**	**Total time from interest to set up (weeks)**	**Time from set up to screening (weeks)**	**Time from set up to randomization (weeks)**
UK (15 sites)	8.5	13.3	42.0	6	30.9
Belgium (8 sites)	15.6	14.8	38.0	16	51.0
Estonia (1 site)	5	4	84	6	9
Germany (1 site)	5	8	56	-	-
Greece (5 sites)	4.0	7.2	43.0	11	12.8
Hungary (1 site)	4	14	48	7	27
India (1 site)	2.0	13.0	104.0	7	-
Ireland (1 site)	7	50	60	18	30.0
Italy (3 sites)	10.5	27.0	77.0	4	13
Russia (1 site)	6	29	64	20	79
**All centers**	**6.8**	**18.0**	**61.6**	**11**	**31.6**

### Patient eligibility

Sixteen per cent of screened TBI patients with ICP monitoring were eligible. The Eurotherm3235Trial inclusion criteria are tighter than most previous trials and the pilot shows they have been effective at recruitment of a homogenous group of TBI patients with brain swelling (Table 2). The most common reasons for exclusion were: ICP less than 20 mmHg, ‘other’ reason, age over 65 years and more than 72 hours from injury (Table 3).

**Table 2:** Marshall classification for abnormalities seen in computerized tomography scan of the brain

	**N**	**%**
Total number of	67	100
patients randomized		
Missing	1	1.4
Diffuse Axonal Injury, grade I	0	0.0
Diffuse Axonal Injury, grade II	10	14.9
Diffuse Axonal Injury, grade III	12	17.9
Diffuse Axonal Injury, grade IV	5	7.4
Evacuated mass lesion	21	31.3
Non-evacuated mass lesion	18	29

**Table 3:** Reasons for exclusion during the pilot phase of the Eurotherm3235Trial

**Reason for exclusion**	**Number of patients**	**Percentage**
ICP <20 mmHg	138	40
Other reason* (including decompressive craniectomy)	46	13
Age of patient	42	12
> 72 hours from initial head injury	41	12
Unlikely to survive 24 hours	20	6
Patient receiving induced hypothermia	12	4
Administration of barbiturate infusion	12	3
Temperature <34.0°C at hospital admission	11	3
No relative consent	7	2
Open traumatic head injury	8	2
No cooling device	5	1
Core temperature <36.0°C at randomization	4	1
Normal CT scan	2	1
Pregnancy	1	0
**Total**	**349**	**100**

*The category other includes decompressive crainiotomy (largest group), enrolled in another trial, no research staff available, multiple trauma, no relatives available, no permanent address, severe burns. *CT* computerized tomography, *ICP* intracranial pressure.

### Feasibility of protocol

Assessment of delivery of cooling showed that, of 34 patients randomized to hypothermia, the average time to reach target temperature was 4 hours (range 1 to 12 hours). Patients remained within the target range of 32 to 35°C for an average of 86 hours (range 5 to 153 hours). One patient did not consistently achieve hypothermia (≤35°C) and four patients (12%) did not receive the minimum 48 hours of hypothermia therapy. The temperature of eight patients (24%) overshot the lower target limit of <32°C within the first 3 days of hypothermia therapy with the temperature of one patient recorded at 30°C because of problems with the cooling device used. In summary, we found an 88% compliance rate with the trial protocol for therapeutic hypothermia.

Two out of 33 patients crossed over from the control to the hypothermia group. In one, the reason was a clinician decision based on the patient’s condition. In the other, it was uncertainty of the trial team in randomizing their first patient. There were seven serious or severe adverse events (Table 4), all unrelated to the intervention. It should be noted that death is not unexpected in this patient cohort and a mortality of 30% is usual [10,13,14].

**Table 4:** Serious adverse events (SAE)

1. Hospitalization	Bleeding - defined as a new haemorrhage requiring 2 units of packed red cells	Unrelated
2. Hospitalization	Bleeding - defined as a new hemorrhage requiring 2 units of packed red cells	Unrelated
3. Life threatening	Cerebral perfusion pressure <50 mmHg for 15 minutes	Unrelated
4. Death	Cardiovascular instability - systolic blood pressure <90 mmHg for 30 minutes	Unrelated
5. Life threatening and persistent of significant disability or incapacity	Cerebral perfusion pressure <50 mmHg for 15 minutes	Unrelated
6. Death	Cerebral perfusion pressure <50 mmHg for 15 minutes	Unrelated
7. Life threatening	Cerebral perfusion pressure <50 mmHg for 15 minutes	Unrelated

Each line describes an SAE reported during the pilot phase of the Eurotherm3235Trial, and each SAE is classified according to International Conference on Harmonisation, Good Clinical Practice (http://www.ich.org/).

One of the objectives set out in the research contract between ESICM and the University of Edinburgh was to seek funding from another source. We are grateful to the National Institute for Health Research, Health Technology Assessment Programme for funding the full trial. Therefore, the new funder is a UK statutory funding agency.

## Discussion

The pilot phase of the Eurotherm3235Trial ran from January 2009 until August 2011, with the aims of assessing the design and implementation of data collection systems, recruitment and protocol feasibility in the first approximate 50 patients. During this period, 67 patients were actually randomized. The information gained from the pilot has informed refinement and improvement of the trial with regard to management, patient eligibility and trial size.

### Trial management

Based on the invaluable experience obtained from the pilot, a significant number of measures have been put in place to ensure compliance with the trial protocol through improved support of the trial sites. Study site initiation, access to support and feedback have all been refined.

Each site receives a complete Investigator Site File, Chief Investigator presentation on DVD and print-outs of all trial related documentation (including a user guide for the eCRF). These are delivered by courier prior to their initiation web-meeting. All UK sites have received a site initiation visit by one of the Trial Managers. In most cases, non-UK sites undertake an initiation meeting with one of the Trial Managers by web conference using an online ‘Meeting Room’ set up by our IT department. In rare circumstances a site visit will be conducted if specifically asked for by the investigator at the site (for example, because of language difficulties). This has reduced the carbon footprint of the trial significantly.

After set up, weekly emails are sent to all database users to inform them of any patients randomized during the week. A member of the trial team is also available in the trial office to support the sites, answering phone calls/queries and a 24-hour helpline is now available. This is held by a member of the trial team on an on-call basis, the number is on the trial website.

Each time a patient is randomized, the Trial Manager responsible for the site will contact the local team personally soon after randomization to thank them for randomizing the patient and offer support and advice if required at any time. Four weeks after randomization, all data in the core eCRF forms (day 1 to 7 forms, Modified Oxford Handicap scale and follow-up forms) are checked for missing data or errors in the eCRF by the Trial Manager responsible for the site. At this time queries are raised and feedback provided to the local team. When the forms have been completed satisfactorily they are frozen in the eCRF by the Data Manager.

This system allows the Trial Manager to identify whether the site has collected the data and if there are any issues which need to be resolved or require further training. Some sites have been unsure how to complete the forms in the eCRF for their first patient; therefore, by receiving feedback from the Trial Manager, they learn how to use the eCRF for their subsequent patients. This has improved data quality within the eCRF.

An annual Investigator Meeting is held in the autumn each year and all recruiting teams are invited to attend. This coincides with a large European meeting that many investigators already attend. A Eurotherm3235Trial Newsletter is produced by the trial team every 2 months and is uploaded to the trial website (http://www.eurotherm3235trial.eu/project/index.phtml). This gives information on recruitment, new sites, conference presentations and important trial updates. The newsletter link is sent by email to all sites that have registered interest along with all initiated sites.

### Patient eligibility

During the internal pilot, 416 patients with ICP monitoring were screened across the 37 trial sites. Of these, 67 (16%) fulfilled the requirements for randomization. During protocol development, the predicted ratio of screened to eligible patients for the Eurotherm3235Trial internal pilot was derived from an observational study that recruited 201 patients managed in “good” sites. These data led us to believe that 50% of all ICP monitored patients would be eligible for recruitment, that is develop ICP >20 mmHg despite simple measures, often described as “stage I” treatments [11].

Two of the most frequent reasons for exclusion from the trial were age over 65 years and more than 72 hours from injury (Table 2). As a result of this analysis, recruiting sites and all database users were surveyed and asked if they would be prepared to randomize older patients and those more than 72 hours from injury. Following a majority positive response, we submitted a protocol amendment to every ethics committee involved in the trial to remove the upper age limit for inclusion (allowing the clinician at each site to triage patients) and increase the time from injury from 72 hours to 10 days. This change was implemented in January 2012.

### Trial size

As a result of the internal pilot the sample size for the full trial was reduced to 600 patients. Two factors underpinned this decision: the homogeneity of the patient group - unlike most previous trials all patients had evidence of brain swelling (raised ICP); and we showed an optimised cooling intervention could be delivered, which meant that the meta-analysis by Peterson and colleagues [10] was the most relevant for the power calculation, rather than the median estimate of effect of six meta-analyses on which our original power calculation was based [12].

The heterogeneity of TBI is considered one of the principal barriers to finding effective therapeutic interventions. The Eurotherm3235Trial inclusion criteria are tighter than most previous trials and have been effective at recruitment of a more homogenous group of TBI patients with brain swelling. This is potentially the subgroup of TBI patients most likely to benefit from titrated hypothermia [15-17]. The cooling intervention in this trial is optimized by rapidly inducing hypothermia using 20 to 30 ml/kg refrigerated 0.9% sodium chloride and is subsequently managed as suggested by meta-analysis [10,18]. Thus all patients receive the same ICP management without barbiturate infusion throughout the trial period, hypothermia is maintained for a minimum of 48 hours, and rewarming is slow and controlled (1°C/4 hours). The pilot phase has confirmed that we can deliver this cooling protocol and in a less heterogeneous group of TBI patients (that is, only those with brain swelling).

The most relevant recent systematic review is therefore that by Peterson and colleagues [10], which found an overall trend towards 25% improved outcome with hypothermia, in trials of sufficient quality (49.4% versus 41.8% favorable outcome, hypothermia versus control, respectively; relative risk (RR) 1.25; 95% confidence interval (CI) 0.96 to 1.62). In the subset of trials in which hypothermia was continued for >48 hours the effect on outcome was greater (RR 1.91; 95% CI 1.28 to 2.85) reaching statistical significance. When ICP was managed without barbiturate infusions, hypothermia was also associated with a statistically significant improvement in outcome (RR 1.79; 95% CI 1.27 to 2.52). Similarly, hypothermia was also associated with a trend to a 20% reduction in mortality, overall (24.8% versus 28.6% mortality, hypothermia versus control, respectively; RR 0.80; 95% CI 0.59 to 1.09). Mortality was reduced in the subset of studies in which hypothermia was continued for >48 hours (RR 0.51; 95% CI 0.33 to 0.79) or when ICP was managed without barbiturate infusion (RR 0.58; 95% CI 0.40 to 0.85).

With a conventional dichotomous analysis of the GOSE, comparing the proportions of patients with an unfavorable outcome in the two groups, a 600 patient trial has 81% power at the 5% significance level (two-sided) to detect an absolute reduction of 12% (60% reducing to 48%). There is 87% power to detect an absolute reduction of 13% (60% reducing to 47%). This is conservative compared with the results of the systematic review by Peterson and colleagues [10] in which optimized therapeutic cooling was associated with an absolute reduction in the proportion of patients with unfavorable outcome of 26% [10]. Furthermore, using an ordinal analysis of the GOSE together with covariate adjustment there is the potential to increase the statistical efficiency of the analysis. If we achieve the efficiency gains suggested by simulations run by the IMPACT investigators [19] and demonstrated in a reanalysis of the CRASH trial [20], then a trial of 600 patients would have equivalent power to a trial of 1,000 patients. This would give 80% power at the 5% significance level (two-sided) to detect an absolute reduction of 9% (60% reducing to 51%).

By way of comparison “The Prophylactic Hypothermia Trial to Lessen Traumatic Brain Injury (POLAR-RCT)” [21] aims to recruit 500 patients in Australasia to detect a 15% absolute reduction in unfavorable outcome (GOSE at 6 months. This is the only concurrent large trial of hypothermia in TBI, which will answer a different but complementary research question (does prophylactic early hypothermia improve outcome after traumatic brain injury?) to the Eurotherm3235Trial, which is testing titrated hypothermia to reduce ICP and the effect that may have on outcome.

## Conclusion

TBI remains a significant cause of morbidity and mortality in the world. The options to manage raised ICP remain limited and each are associated with significant risk. Therapeutic hypothermia is already used in many centers around the world as an option in the management of patients with severe TBI and therefore it is essential to confidently confirm or refute outcome benefit, rather than merely palliation of intracranial hypertension. In the Eurotherm3235Trial pilot we have demonstrated that we can effectively manage a large international multicenter randomized controlled trial of hypothermia (32 to 35°C) to control ICP. Site initiation has proceeded well, online randomization and data collection are established and the cooling protocol can be delivered. Crucially, the pilot has demonstrated recruitment of a homogeneous patient group and allowed a robust estimate of sample size. This has been important in helping to achieve support from the NHS National Institute for Health Research Health Technology Assessment Program which will fund the trial to completion in 2016. As of now (20 March 2013) over 1,256 patients have been screened and 210 have been randomized (http://www.eurotherm3235trial.eu).

## Abbreviations

CI: Confidence interval; DSMB: Data safety monitoring board; eCRF: Electronic case report form; GOSE: Extended glasgow outcome scale; ICP: Intracranial pressure; RR: Relative risk; TBI: Traumatic brain injury.

## Competing interests

The authors declare that they have no competing interest.

## Author information

Chief Investigator: Professor Peter Andrews.

## Authors’ contributions

PJDA, GM, BH: trial design, protocol writing, data analyses and manuscript preparation. LHS, CGB: protocol writing, data analyses and manuscript preparation. MJB: data analyses and manuscript preparation. JKJR: data analyses and manuscript preparation. DDB: protocol writing, data analyses and manuscript preparation and member of the “Research, Scientific and Financial Committee”. The Eurotherm3235Trial collaborators: assessment of feasibility of eCRF, screening and recruitment. All authors have read and approved the final manuscript.
